# Genomic and antimicrobial susceptibility characterization of a clinical *Pasteurella aerogenes* isolate from a human patient

**DOI:** 10.3389/fcimb.2026.1900215

**Published:** 2026-09-30

**Authors:** Ying Chen, Gerrit Brandis, Hong Li, Yongjun Luo, Huiyu Wang, Jinrong He, Yumeng Wang

**Affiliations:** 1 Laboratory Department, Qionghai People’s Hospital, Qionghai, Hainan, China; 2 Department of Cell and Molecular Biology, Uppsala University, Uppsala, Sweden; 3 Xinjiang Uygur Autonomous Region Center for Disease Control and Prevention, Urumqi, China; 4 National Key Laboratory of Intelligent Tracking and Forecasting for Infectious Disease, National Institute for Communicable Disease Control and Prevention, Chinese Center for Disease Control and Prevention, Beijing, China

**Keywords:** antimicrobial susceptibility, function analysis, genomic features, One Health, *Pasteurella aerogenes*, whole genome sequencing

## Abstract

**Background:**

*Pasteurella aerogenes* is an opportunistic zoonotic pathogen associated with reproductive disorders in animals and sporadic human infections. However, the complete genome, antimicrobial susceptibility profile, and phylogenetic relationships of clinical *P. aerogenes* isolates, particularly from China, remain poorly understood.

**Methods:**

We sequenced the complete genome of the clinical *P. aerogenes* isolate 21PA20240911 from a patient with urinary tract infection in Hainan, China, using a hybrid PacBio and Illumina sequencing approach. Comparative genomic analyses, including genome feature characterization, ANI-based species delineation, virulence gene screening, antimicrobial susceptibility testing, and cgSNP-based phylogenetic analysis, were performed to investigate the strains genomic landscape and evolutionary origin.

**Results:**

The genome of strain 21PA20240911 was 2.3Mbp with a G+C content of 40.84%. ANI analysis confirmed its taxonomic assignment as *P. aerogenes* (97.61% identity with the reference strain). The strain carried 28 virulence-associated genes. Notably, *tviB* and *wbpD* were detected only in our strain and a duck-derived Sichuan isolate. The isolate was resistant only to erythromycin but showed nonsusceptibility to multiple β-lactams and tetracyclines. Resistance-associated determinants included *tet (B)/tetR* and a putative PBP3/FtsI-like protein variant carrying D350N and S357N substitutions. Phylogenetic analysis tentatively suggests the possibility of zoonotic transmission from local poultry for this strain.

**Conclusion:**

This study provides the first complete genome sequence of a clinical *P. aerogenes* isolate from China, offering a genomic foundation for understanding its pathogenicity and antimicrobial resistance. From a One Health perspective, enhanced surveillance in both clinical and veterinary settings is warranted.

## Introduction

*Pasteurella aerogenes* (*P. aerogenes*), a Gram-negative, facultatively anaerobic, short rod-shaped bacterium of the family *Pasteurellaceae*, is recognized as an opportunistic zoonotic pathogen (Hommez and Devriese, 1976). It is commonly considered part of normal intestinal microbiota of swine (Koneman et al., 1997),but under certain conditions it can act as a pathogen in both animals and humans.

In veterinary medicine, *P. aerogenes* has been associated with reproductive and systemic infections in multiple animal hosts, including abortion in swine (Fodor et al., 1991) and rabbits (Thigpen et al., 1978), as well as infections in poultry (Yuan et al., 2022) and hamsters (Freeman et al., 2004). In humans, although infections are relatively rare, *P. aerogenes* has been implicated in clinically significant conditions such as stillbirth associated with occupational exposure to swine (Thorsen et al., 1994), vertebral osteomyelitis in immunocompetent individuals (Quiles et al., 2000), and infections of the urinary tract, wounds, and ulcers (Ejlertsen et al., 1996; Alaygut and Engin, 2018). Human infections are most frequently linked to direct or indirect contact with pigs, such as pig bites or occupational exposure (Barnham, 1988; Ejlertsen et al., 1996), although cases without clear animal exposure have also been reported (Quiles et al., 2000).

From a clinical perspective, *P. aerogenes* infections are generally treatable with β-lactam antibiotics, with penicillin considered the first-line therapy (Stadler, 2024). Alternative therapeutic options include ampicillin, amoxicillin-clavulanate, cephalosporins, trimethoprim-sulfamethoxazole, doxycycline, and fluoroquinolones (Goldstein et al., 2001; Cross, 2025). However, increasing reports of antimicrobial resistance in *P. aerogenes* have raised clinical concerns. Multidrug-resistant isolates have been identified in both animal and human sources, including strains resistant to multiple antibiotic classes and carrying mobile resistance determinants such as ESBL genes and colistin resistance genes (Keeratikunakorn et al., 2024; D et al., 2022; MEI et al., 2012). These findings suggest that *P. aerogenes* may represent an emerging reservoir of antimicrobial resistance with potential zoonotic implications.

In addition to its clinical relevance, the taxonomy and phylogenetic placement of *P. aerogenes* and related porcine *Pasteurellaceae* warrant further investigation. The *P. aerogenes*–*Pasteurella mairii* (*P. mairii*)–*Actinobacillus rossii* (*A. rossii*) complex has historically been difficult to identify because of overlapping phenotypic characteristics. *P. aerogenes*, *P. mairii*, and *A. rossii* were recognized as genetically distinct but closely related taxa on the basis of 16S rRNA gene sequence comparison and DNA- DNA hybridization (Christensen et al., 2005). With the increasing availability of genome sequences, genome-based taxonomic approaches may provide complementary evidence for assessing species-level relationships within the *Pasteurellaceae* (Richter and Rosselló-Móra, 2009; Kim et al., 2014; Christensen et al., 2020; Riesco and Trujillo, 2024).

Despite increasing reports of *P. aerogenes* in veterinary and human infections, data on clinical isolates remain limited. Here, we characterize a clinical *P. aerogenes* isolate recovered from a urinary tract infection case in Hainan, China. This study expands current knowledge of this under-characterized *Pasteurellaceae* species and provides insights into its clinical and biological characteristics.

## Materials and methods

### Clinical information and species identification of strains

A 67-year-old male farmer was admitted on September 10, 2024, due to a 7-day history of difficulty urinating. The patient experienced unexplained straining, a weakened urinary stream, and prolonged voiding time, accompanied by frequency, urgency, and dysuria, with nocturia 4-5 times/night. No fever or gross hematuria was reported. Prior to admission, urinary ultrasonography at an outside hospital revealed benign prostatic hyperplasia (approximately 38 × 33 mm) with calcification. On admission, digital rectal examination showed a grade II enlarged prostate with firm texture, smooth surface, no tenderness, and a shallow median sulcus. Subsequent urinary ultrasound and CT examination demonstrated increased post-void residual volume (549 mL), consistent with urinary retention. Urine specimens were collected by midstream clean-catch sampling strictly in accordance with the midstream clean-catch collection procedure specified in Clause 5.2 of WS/T 489-2024, Guidelines for the testing of urine specimens in the clinical microbiology laboratory, a health industry standard of the People’s Republic of China (National Center for Clinical Laboratories, 2024). Laboratory testing revealed markedly abnormal urinalysis: turbid urine, white blood cells 112/μL, red blood cells 539.10/μL, bacterial count >25,000/μL, and urinary smear showed a large number of Gram-negative bacteria. Bacterial species identification and biochemical profiling were performed using the VITEK MS and VITEK 2 systems (bioMérieux, France), respectively, following the manufacturer’s standard protocols. The patient was clinically diagnosed with benign prostatic hyperplasia, urinary retention, and urinary tract infection. The patient received ceftazidime for antimicrobial treatment, and repeat urine culture three days later showed no bacterial growth.

### Ethics statement

The study was reviewed and approved by the Medical Ethics Committee of Qionghai People’s Hospital (Approval No. QH-KY-2026004). The requirement for informed consent was waived because this retrospective study used laboratory data only and did not involve patient privacy or data-security concerns. No identifiable personal information is included in this manuscript.

### Antimicrobial susceptibility testing and β-lactamase detection

Antimicrobial susceptibility testing of the clinical *P. aerogenes* isolate was performed Kirby-Bauer (KB) disk diffusion, broth microdilution, and E-test methods, according to the antimicrobial agent tested. Disk diffusion testing was performed on Mueller-Hinton agar supplemented with 5% sheep blood (MH-B agar). Broth microdilution was used to determine the minimum inhibitory concentrations (MICs) of selected agents, and E-test strips (Liofilchem, Italy) were used for additional MIC determination on MH-B agar. Three biological replicates were performed for each assay. *Streptococcus pneumoniae* ATCC 49619 was included as a quality-control strain.

For antimicrobial agents with available criteria, results were interpreted using the CLSI M45, 3rd edition (2016), *Pasteurella* spp. criteria as comparator-based breakpoints. Because species-specific CLSI breakpoints are not available for *P. aerogenes*, these interpretations were considered tentative. For agents without an applicable CLSI M45 breakpoint, only the raw inhibition-zone diameters, MICs, or E-test results were reported, without categorical susceptibility interpretation. The antibiotic profiles, raw susceptibility results, applicable breakpoint references, and interpretations are summarized in **Table 1** (Clinical and Laboratory Standards Institute, 2016).

**Table 1:** Antimicrobial susceptibility profile and resistance genes of the clinical *P. aerogenes* isolate 21PA20240911.

Antimicrobial agent	KB method (mm)	MIC (μg/mL)	E-Test (μg/mL)	Comparator breakpoint used	Interpretation (S/I/R)	Genotypic AMR determinant(s) detected
β-Lactams						
Ampicillin	26 (10μg)	2	–	*Pasteurella* spp., CLSI M45 Ed3: disk S ≥27 mm; MIC S ≤0.5	Nonsusceptible	PBP3/FtsI-like protein variant (D350N, S357N; RGI/CARD annotation*)*
Penicillin	12 (10μg)	>8	–	*Pasteurella* spp., CLSI M45 Ed3: disk S ≥25 mm; MIC S ≤0.5	Nonsusceptible	PBP3/FtsI-like protein variant (D350N, S357N; RGI/CARD annotation*)*
Amoxicillin-clavulanate	–	1/0.5	–	*Pasteurella* spp., CLSI M45 Ed3: MIC S ≤0.5/0.25	Nonsusceptible	PBP3/FtsI-like protein variant (D350N, S357N; RGI/CARD annotation*)*
Ceftriaxone	38 (30μg)	<0.12	–	*Pasteurella* spp., CLSI M45 Ed3: disk S ≥34 mm; MIC S ≤0.12	S (Susceptible)	–
Cefuroxime	–	0.5	–	No applicable breakpoint	Not interpreted	–
Cefoperazone	27 (75μg)	–	–	No applicable breakpoint	Not interpreted	–
Ceftazidime	28 (30μg)	–	–	No applicable breakpoint	Not interpreted	–
Cefazolin	27 (30μg)	–	4	No applicable breakpoint	Not interpreted	–
Cefalexin	29 (30μg)	–	–	No applicable breakpoint	Not interpreted	–
Imipenem	30 (10μg)	–	–	No applicable breakpoint	Not interpreted	–
Piperacillin	29 (100μg)	–	–	No applicable breakpoint	Not interpreted	–
Ceftobiprole	–	–	0.012	No applicable breakpoint	Not interpreted	–
Cefotaxime	–	–	0.064	No applicable breakpoint	Not interpreted	–
Cefiderocol	–	–	<0.016	No applicable breakpoint	Not interpreted	–
Macrolides						
Erythromycin	21 (15μg)	4	–	*Pasteurella* spp., CLSI M45 Ed3: disk S ≥27, I 25–26, R ≤24 mm; MIC S ≤0.5, I 1, R ≥2	R (Resistant)	–
Azithromycin	29 (15μg)	–	–	*Pasteurella* spp., CLSI M45 Ed3: disk S ≥20 mm	S (Susceptible)	–
Phenicols						
Chloramphenicol	32 (30μg)	–	–	No verified applicable breakpoint in the present dataset	S (Susceptible)	–
Tetracyclines						
Tetracycline	9 (30μg)	–	–	*Pasteurella* spp., CLSI M45 Ed3: disk S ≥23 mm	Nonsusceptible	–
Doxycycline	17 (30μg)	4	–	*Pasteurella* spp., CLSI M45 Ed3: disk S ≥23 mm; MIC S ≤0.5	Nonsusceptible	*tet*(B)*, tetR*
Minocycline	26 (30μg)	–	0.125	No applicable breakpoint	Not interpreted	–
Folate pathway inhibitors						
Trimethoprim-sulfamethoxazole	44 (1.25/23.75μg)	<0.5/9.5	–	*Pasteurella* spp., CLSI M45 Ed3: disk S ≥24 mm	S (Susceptible)	–
Fluoroquinolones						
Levofloxacin	32 (5μg)	0.19	–	*Pasteurella* spp., CLSI M45 Ed3: disk S ≥28 mm; MIC S ≤0.06	Discordant: susceptible by KB; nonsusceptible by MIC	–
Moxifloxacin	–	1	–	*Pasteurella* spp., CLSI M45 Ed3: MIC S ≤0.06	Nonsusceptible	–
Norfloxacin	31 (10μg)	–	–	No applicable breakpoint	Not interpreted	–
Ciprofloxacin	33 (5μg)	–	0.094	No applicable breakpoint	Not interpreted	–
Others						
Polymyxin B	18 (300iu)	–	–	No applicable breakpoint	Not interpreted–	–
—	—	—	–		—	*E. coli* EF-Tu mutants

All breakpoints were derived from CLSI M45, 3rd edition, Table 17 (*Pasteurella* spp.), where available. Because species-specific CLSI breakpoints for *P. aerogenes* are unavailable, these interpretations are comparator-based. “Nonsusceptible” indicates that the result did not meet the available susceptible-only criterion; it should not be interpreted as “resistant” when no resistant breakpoint is defined.

† The detected PBP3/FtsI-like protein was annotated by RGI/CARD as a Haemophilus influenzae PBP3 protein variant model and carried D350N and S357N substitutions. Its specific contribution to the observed beta-lactam nonsusceptibility was not experimentally confirmed.

β-Lactamase production was assessed using a nitrocefin-based test, as previously described (Mugg, 1983), with three biological replicates.

### DNA preparation and whole-genome sequence assembly

Genomic DNA was extracted from overnight culture Columbia Blood Agar of *P. aerogenes* isolate using a DNA extraction kit following the manufacturer’s instructions (No.69504, QIAGEN, Germany). The Qubit 2.0 Fluorometer (Life Technologies, Carlsbad, California, USA) was used for genomic DNA quantification. Qualified DNA was used to construct PacBio and Illumina libraries, which were sequenced on the PacBio Sequel II and Illumina NovaSeq PE150 platforms, respectively, at Beijing Novogene Bioinformatics Technology Co., Ltd. Low-quality PacBio reads were filtered using SMRT Link v8.0, and the filtered long reads were assembled denovo with Canu v2.0 (https://github.com/marbl/canu) to generate the initial assembly (Koren et al., 2017). The initial assembly was polished through three rounds of Racon v1.4.13 using PacBio long reads (Vaser et al., 2017), followed by three rounds of Pilon v1.22 polishing using Illumina short reads (Walker et al., 2014). Detailed parameters are provided in **Supplementary Table S1**. Genome completeness and contamination were assessed using CheckM (Parks et al., 2015).

### Antibiotic resistance genes and virulence genes prediction

The RGI program (version 6.0.3) was employed to predict antimicrobial resistance genes in the genome using default parameters (Jia et al., 2017; Alcock et al., 2023). Additionally, putative virulence-associated genes were identified by screening the predicted gene sequences against the Virulence Factor Database (VFDB) (Chen et al., 2005; Zhou et al., 2025). Hits with ≥70% nucleotide sequence identity and ≥70% alignment coverage were retained for downstream analysis, following a published ABRicate–VFDB workflow (Xie et al., 2024). Matches failing either criterion, including partial matches with <70% coverage, were excluded. Retained hits are reported as putative virulence-associated homologues, and their sequence identity and coverage are listed in **Supplementary Table S1**, the results were visualized using the tvBOT online program (Xie et al., 2023).

### Genomic data collection and average nucleotide identity analysis

Twenty *P. aerogenes* genomes were retrieved from the NCBI GenBank database on January 10, 2026, including the porcine reference genome NCTC 13378 and 19 animal-derived genomes. For species-level genomic relatedness assessment by ANI, two *P. mairii* and eight *A. rossii* genomes were additionally included because these taxa comprise the historically recognized *P. aerogenes*–*P. mairii*–*A. rossii* complex and show overlapping phenotypic characteristics (Christensen et al., 2005; Riesco and Trujillo, 2024). The comparison was focused on this complex because preliminary ANI screening of representative *Pasteurella sensu stricto* genomes, including *Pasteurella multocida* ATCC 43137, showed less than 80% ANI with the included taxa (**Supplementary Figure S1**). Detailed genome information is provided in **Supplementary Table S1**.

ANI values for the 31 strains (21 *P. aerogenes* strains, 8 *A. rossii* strains and 2 *P. mairii* strains) were calculated using fastANI (v1.33) (Jain et al., 2018). Hierarchical clustering based on ANI values was performed using the UPGMA method via the Python SciPy package (Virtanen et al., 2020).

### Genomic composition, functional annotation, and visualization

The assembled genome of strain 21PA20240911 was used to predict coding genes, repetitive sequences, non-coding RNAs, genomic islands, transposons, prophages, and CRISPRs. Coding genes were predicted using GeneMarkS v4.17. Interspersed and tandem repeats were identified using RepeatMasker open-4.0.5 and TRF v4.07b, respectively (Benson, 1999). tRNA and rRNA genes were predicted using tRNAscan-SE v1.3.1 and rRNAmmer v1.2, respectively (Lagesen et al., 2007). Small RNAs were annotated against Rfam release 14.10 and confirmed using cmsearch v1.1rc4 with default parameters (Gardner et al., 2009; Nawrocki et al., 2009).Genomic islands were predicted using IslandPath-DIOMB v0.2 (Hsiao et al., 2003), and transposons were identified using TransposonPSI. Prophages and CRISPR loci were predicted using PhiSpy v2.3 (Akhter et al., 2012) and CRISPRdigger v1.0, respectively.

Gene functions were annotated against the GO (Gene Ontology; release 2023-11-15) (Ashburner et al., 2000), KEGG (Kyoto Encyclopedia of Genes and Genomes; release 2024-03) (Kanehisa et al., 2004), COG (Clusters of Orthologous Groups; COG2020) (Galperin et al., 2015), bacterial NR (Non-Redundant Protein Database; release 2024-02-07), and TCDB (Transporter Classification Database; release 2024-07-29) (Saier et al., 2021) databases. Predicted proteins were searched against these databases using BLASTp. Hits with an E-value <1×10^-5^, sequence identity ≥40%, and query coverage ≥40% were retained, and the highest-scoring qualified hit was assigned as the functional annotation. CAZymes were annotated against CAZy v12 (release 2023-08-03) (Cantarel et al., 2009).

Circos v0.69-8 was used to visualize the assembled genome of strain 21PA20240911 together with genome composition and functional annotation results (Krzywinski et al., 2009). For comparative genomic visualization, strain NCTC13378 (GCA_900637275.1) was used as the reference genome. Homologous regions between 21PA20240911 and NCTC13378 were identified using BLASTn Blast+v2.15.0 with an E-value threshold of 1×10^-10;^ hits shorter than 500 bp with less than 95% nucleotide identity were discarded to exclude spurious matches. Strain-specific genes were identified by reciprocal best-hit BLASTP analysis (E-value ≤ 1×10^-5^). The comparative circular map displayed chromosome backbones, GC content, GC skew, COG and KEGG annotation distributions, tRNA/rRNA loci, defense-related genes (COG category V), and strain-specific genes. For details, see the **Supplementary Material 10**.

## Methods

### Core-pan genome analysis and core-genome maximum-likelihood phylogenies

Core- and pan-genome analyses were performed using Roary v3.11.2 with the -e -n -s -ap options on 21 *P. aerogenes genomes*, including strain 21PA20240911 and 20 publicly available genomes (Page et al., 2015). Core genes were defined as gene clusters present in ≥99% of genomes (980 clusters; default -i 95 and -cd 99) and were aligned using MAFFT v7.520. SNPs were extracted from the core-gene alignment using SNP-sites, and pairwise SNP distances were calculated using a custom Python script (Page et al., 2016). The resulting pairwise core-genome SNP-distance matrix is provided in **Supplementary Table S3**. To evaluate the robustness of the inferred distance relationships, the corresponding estimates from the core-SNP analysis, Gubbins recombination-filtered analysis, and Snippy reference-mapping analysis were compared (**Supplementary Table S2**). A maximum-likelihood phylogenetic tree was reconstructed using IQ-TREE v1.6.12 with the ModelFinder-selected substitution model and 1,000 ultrafast bootstrap replicates (Katoh and Standley, 2013.;Nguyen et al., 2015; Kalyaanamoorthy et al., 2017; Hoang et al., 2018). Recombination filtering with Gubbins v3.4.3 and an independent Snippy v4.6.0 read-mapping analysis were conducted as validation analyses (Croucher et al., 2015). Detailed parameters, scripts, alignments, and validation outputs are provided in the **Supplementary Materials Data 14**.

### Whole-genome data availability

The sequencing data generated in this study have been deposited under NCBI BioProject accession PRJNA1425987.

## Results

### Identification of *P. aerogenes* isolate 21PA20240911

During the clinical evaluation, both *P. aerogenes* and *Escherichia coli* were repeatedly recovered from urine cultures and were identified by VITEK MS. Repeated recovery of *P. aerogenes* from this symptomatic patient supported its clinical relevance. The *P. aerogenes* isolate was selected for further characterization because of its rarity and was designated strain 21PA20240911. After 24 h of incubation at 35°C on 5% sheep blood agar, strain 21PA20240911 formed smooth, grayish-white, circular, moist, translucent colonies measuring 1.5–2.0 mm in diameter (**Figure 1A**). Gram staining at ×1,000 magnification showed Gram-negative coccobacilli, appearing singly or in short chains (**Figure 1B**). The isolate was further identified as *P. aerogenes* using the VITEK 2 system, and its complete biochemical characteristics are presented in **Table 2**.

**Figure 1:**
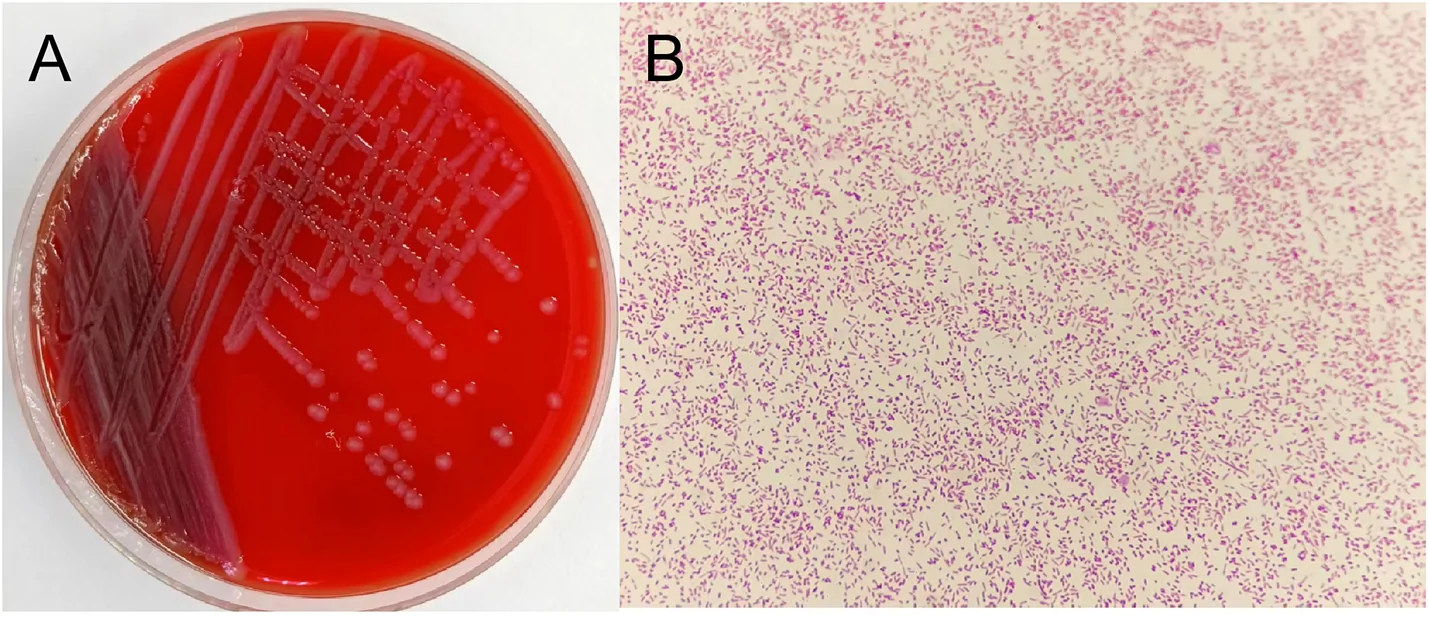
Colony morphology and Gram-staining characteristics of *P. aerogenes* 21PA20240911. **(A)** Colony morphology of *P. aerogenes* on 5% sheep blood agar (24 h, 35 °C). **(B)** Gram-negative coccobacilli of *P. aerogenes* under light microscopy (1000×).

**Table 2:** Biochemical characteristics of the *P. aerogenes* strain 21PA20240911.

Substrate	Reaction	Substrate	Reaction	Substrate	Reaction
APPA	–	ADO	–	PyrA	–
H2S	–	BNAG	–	AGLTp	–
BGLU	–	dMAL	–	dMAN	–
ProA	–	LIP	–	PLE	–
SAC	+	dTAG	–	dTRE	–
ILATk	–	AGLU	–	SUCT	–
GlyA	–	ODC	+	LDC	–
O129R	–	GGAA	–	IMLTa	–
BGAL	–	OFF	–	BALap	–
PHOS	+	BGUR	–	IHISa	–
IARL	–	dCEL	–	ELLM	+
dGLU	+	GGT	+	dSOR	–
dMNE	+	BXYL	–	CMT	–
TyrA	–	URE	+	ILATa	–
CIT	–	MNT	–	5KG	–
NAGA	–	AGAL	–		

### Antimicrobial susceptibility profile and genomic detection of resistance-associated determinants

Antimicrobial susceptibility testing showed that isolate 21PA20240911 was nonsusceptible to ampicillin, penicillin, amoxicillin-clavulanate, tetracycline, doxycycline, levofloxacin, and moxifloxacin, while it was susceptible to ceftriaxone, azithromycin, and trimethoprim-sulfamethoxazole. The isolate was resistant to erythromycin. The isolate was negative for beta-lactamase production. RGI/CARD analysis identified a PBP3/FtsI-like protein variant annotated against the *Haemophilus influenzae* PBP3 reference model, carrying D350N and S357N substitutions, in this beta-lactam-nonsusceptible isolate. In addition, *tet (B)/tetR* were detected in the doxycycline-nonsusceptible isolate. Two EF-Tu variant hits carrying R234F, annotated against an *E. coli* EF-Tu mutant resistance model associated with pulvomycin resistance, were also identified.

For some antibiotics, only specific detection data (inhibition zone diameter or MIC value) were recorded without sensitive/intermediate/resistant classification due to the limited standards for determining the antibiotic sensitivity of *Pasteurella* spp. in the CLSI guidelines. The detailed antimicrobial susceptibility results are presented in **Table 1** to provide a basic reference for the clinical application and subsequent research of this strain.

### Species identification and genomic relatedness analysis

To assess the species-level genomic relatedness of strain 21PA20240911, ANI analysis was performed using genomes of *A. rossii*, *P. mairii*, and *P. aerogenes.* The resulting ANI matrix and hierarchical clustering are shown in **Figure 2A**. Strain 21PA20240911 shared 97.01%–99.87% ANI with other *P. aerogenes* strains, exceeding the commonly applied 95% species-level threshold and supporting its assignment to *P. aerogenes*. In contrast, ANI values between strain 21PA20240911 and *P. mairii* and *A. rossii* were 82.12%–84.57% and 74.36%–78.25%, respectively, demonstrating substantially lower genomic relatedness. Consistent with these results, strain 21PA20240911 clustered with other *P. aerogenes* strains in the ANI-based hierarchical clustering analysis.

**Figure 2:**
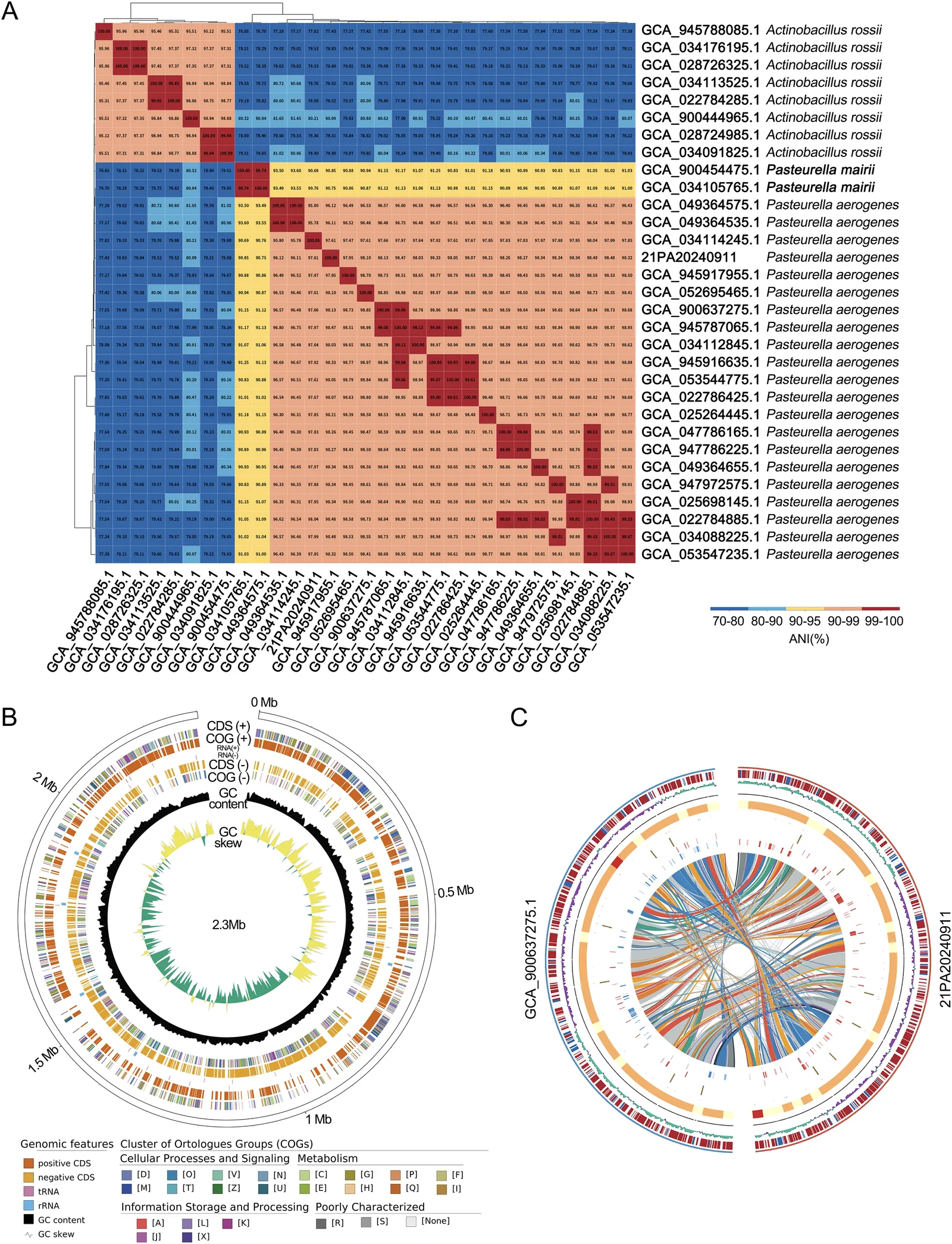
Genomic characterization of strain 21PA20240911. **(A)** ANI-based phylogenetic heatmap. Color gradient indicates pairwise ANI values (%); red = high identity, blue = low identity. Hierarchical clustering trees on both sides show evolutionary relationships. **(B)** Circular genome map of strain 21PA20240911. Tracks from outermost to innermost: (i) genes on the positive and negative strands colored by COG functional categories; (ii) GC content; (iii) GC skew. The central value indicates genome size (2.3 Mbp). Legends at the bottom denote genomic elements and the 21 COG categories. Regional variations in GC skew support the predicted origin (*ori*) and terminus (*ter*) of replication. **(C)** Comparative Circos map of strain 21PA20240911 and reference GCA_900637275.1. Outer to inner tracks show chromosome backbone, GC content, GC skew, COG distribution, KEGG density, tRNA/rRNA positions, defense genes, and strain-specific genes. Central BLAST links indicate synteny. Syntenic relationships were shown as central Bézier curves.

### General genomic features of strain 21PA20240911

Whole-genome sequencing, assembly, and annotation of clinical isolate 21PA20240911 revealed a single circular chromosome of 2.3 Mbp with an overall GC content of 40.84%. The PacBio dataset comprised 3.44 Gb of sequence data, corresponding to approximately 1,495× coverage of the final chromosome. The final assembly consisted of one gap-free contig. Illumina short reads mapped back to the final assembly with an overall alignment rate of 98.81%, supporting the consistency of the assembly with the short-read data. CheckM estimated the genome completeness and contamination to be 97.18% and 1.12%, respectively.

A total of 2,210 genes were predicted, comprising 2,146 protein-coding sequences (CDSs), 57 tRNA genes, 6 rRNA genes, and 8 sRNA genes. Additional genomic elements included 138 repeat regions, 2 prophages, and 6 genomic islands. Physicochemical signatures, such as GC content and GC skew, exhibited marked regional variation, allowing prediction of the putative origin and terminus of replication. Functional annotation against the COG database showed that most CDSs were enriched in metabolism, information storage and processing, and cellular processes and signaling. Non-coding RNAs and defense-related genes were evenly distributed across the genome (**Figure 2B**). Comprehensive multi-database annotation was further performed: 2,143 chromosomal genes were annotated via the NR database; 124 CAZymes were predicted, consisting of 18 CBMs, 6 CEs, 57 GHs and 50 GTs. TCDB identified 411 transporter genes, and PHI-base predicted 412 PHIs. KEGG and GO annotation further characterized metabolic pathways and functional profiles of the strain (**Figures 3A–E**).

**Figure 3:**
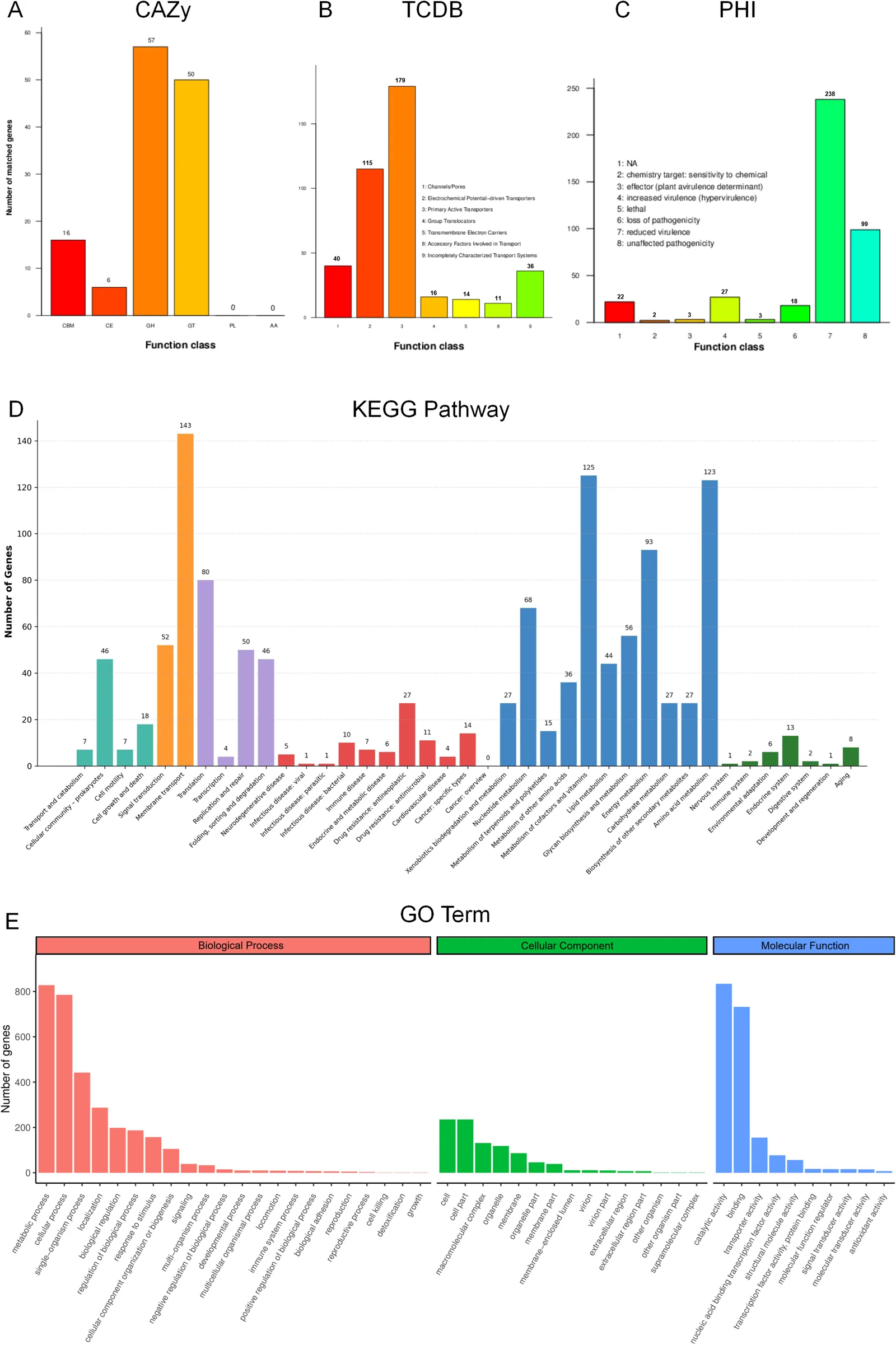
Multi-database functional annotation of strain 21PA20240911. **(A)** CAZymes functional classification. **(B)** TCDB classification of transporter-related genes. **(C)** PHI-base annotation of pathogen-host interaction genes. **(D)** KEGG pathway classification. **(E)** GO classification, showing three main categories: biological process, cellular component, and molecular function.

### Comparative genomic analysis of strain 21PA20240911 against related *P. aerogenes* genomes

To investigate genetic variation, strain-specific genes and functional divergence, a multi-layer comparative genomic Circos plot was constructed between strain 21PA20240911 and the reference strain GCA_900637275.1 (**Figure 2C**). Genomic backbone, GC content, GC skew, COG-based gene distribution, KEGG pathway density, tRNA/rRNA positions, defense genes and strain-specific genes were visualized from outer to inner rings. Overall genomic synteny was well-conserved with no large-scale rearrangement, as indicated by central BLAST homology links. Strain 21PA20240911 harbored 29 COG category V (defense mechanism) genes, compared with 27 in the reference strain, with minor differences in their genomic distribution. Strain-specific genes were significantly enriched in pathways related to drug resistance, virulence, and environmental adaptability. Additionally, enrichment was observed in amino acid metabolism and membrane transport pathways, which may collectively contribute to host-microbe adaptation of this clinical isolate.

A total of 34 virulence-associated genes were screened across all 21 strains (**Figure 4**), among which strain 21PA20240911 carried 28 virulence genes. Notably, *tviB* and *wbpD* were uniquely present in 21PA20240911 and one poultry-origin strain from Sichuan (GCA_025264445.1), suggesting strain-specific pathogenic potential.

**Figure 4:**
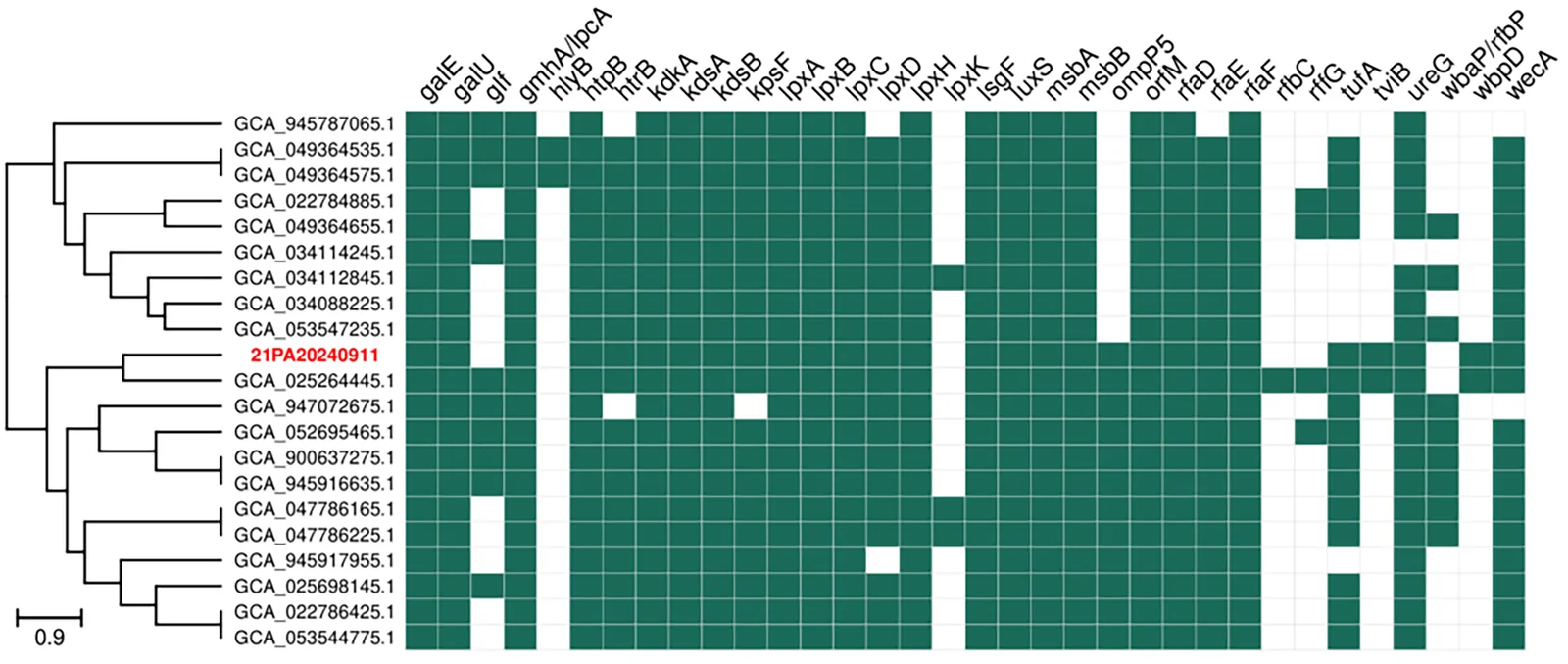
Virulence genes identified among *P. aerogenes* strains: dark green, present; white, absent.

### Pan-genome analysis of *P. aerogenes*

Core-pan genome analysis of 21 *P. aerogenes* strains revealed a total of 4,692 genes within the species pan-genome. Based on their distribution frequency across the strains, these genes were categorized into four groups: 980 core genes (present in all 21 strains), 422 soft-core genes (present in >95% of strains), 897 shell genes (present in 15-95% of strains), and 2,393 cloud genes (present in <15% of strains). Core genes accounted for 20.9% of the total pan-genome.

### Phylogenetic analysis of strain 21PA20240911

Phylogenetic analysis based on cgSNP revealed three major clades (designated I, II, and III). The phylogenetic tree with these clades is shown in **Figure 5**. The study strain 21PA20240911 fell within Clade II, alongside multiple swine-derived and poultry-derived strains, and showed the closer genetic relatedness to strains originating from Sichuan, China (GCA_025698145.1, GCA_025264445.1). The complete pairwise core-genome SNP-distance matrix underlying this analysis is presented in **Supplementary Table S3**.

**Figure 5:**
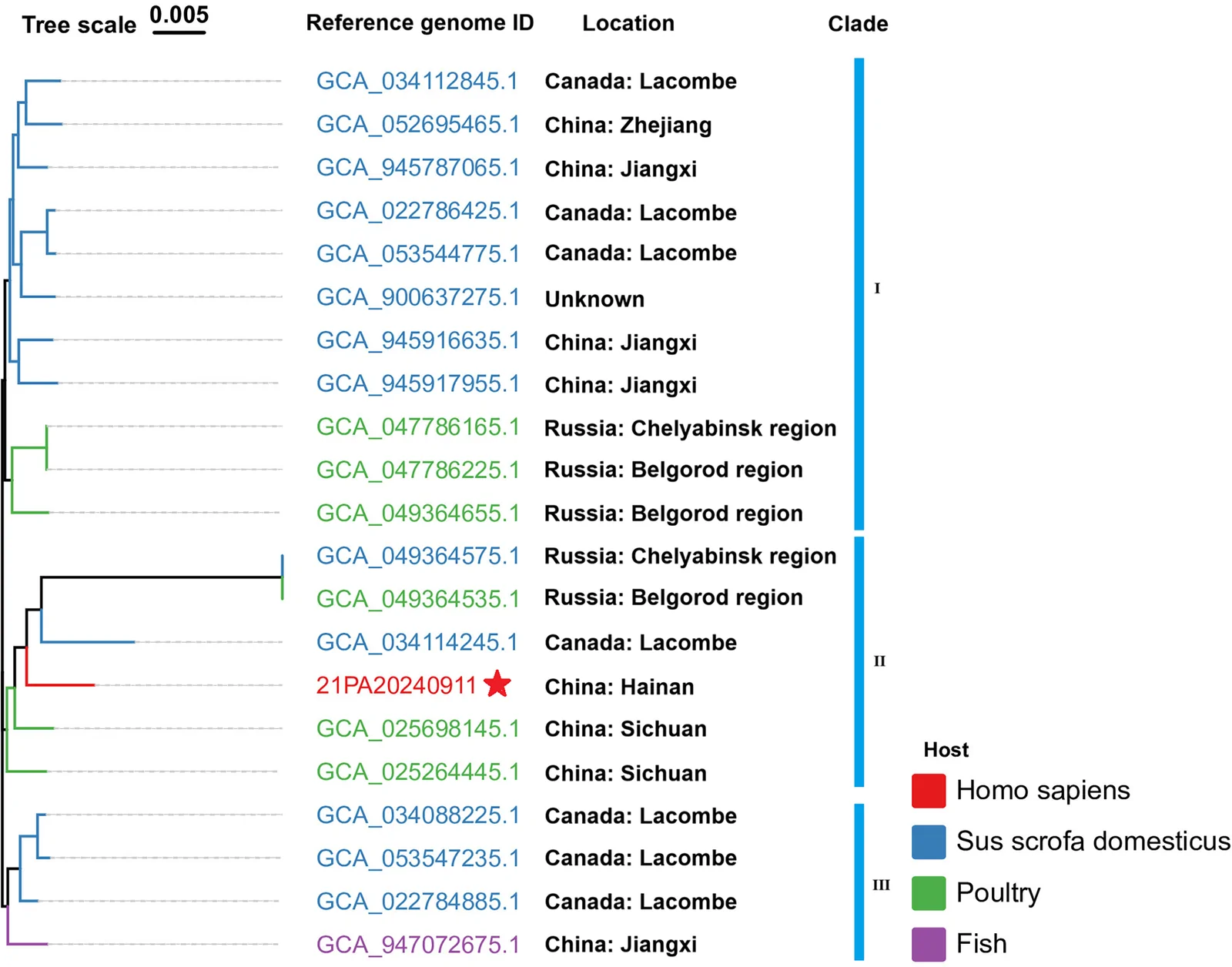
Phylogenetic tree of *Pasteurella aerogenes* strains based on cgSNP analysis. Branch lengths are scaled to substitutions per site (scale bar: 0.005). Colors indicate host origin. Both phylogenetic tree branches and GCA accession numbers are color-coded by host: human, pig, poultry, and fish. The study strain 21PA20240911 (red star) clusters in Clade II with swine- and poultry-derived isolates.

To assess the robustness of the core-genome SNP analysis, we performed validation analyses. Gubbins recombination filtering retained 49,563 polymorphic sites, compared with 55,795 sites in the original SNP alignment. Pairwise distances obtained before and after recombination filtering were highly correlated (Pearson’s r = 0.993), indicating that the overall relationship pattern was stable, although some local branching relationships differed in the recombination-filtered tree (**Supplementary Figure S2**). The independent Snippy read-mapping analysis also showed a concordant distance trend among the examined reference genomes (**Supplementary Figures S3**-**S4**; **Supplementary Table S2**).

## Discussion

In this study, we present the first complete genome sequence of a clinical *P. aerogenes* isolate (21PA20240911) from China, generated using a hybrid PacBio and Illumina sequencing approach. Comparative genomic analysis revealed a unique repertoire of virulence determinants, including the rare *tviB* and *wbpD* genes previously reported only in a duck-derived isolate. Additionally, antimicrobial susceptibility testing and genomic analysis identified the resistance profile and corresponding resistance genes of this strain. These findings provide important insights into the pathogenic potential and genomic characteristics of this understudied opportunistic pathogen.

Notably, *P. aerogenes* and *E. coli* were repeatedly recovered from urine culture obtained during the clinical evaluation. In the context of urinary symptoms, pyuria, marked bacteriuria, abundant Gram-negative bacteria on urine smear, and urinary retention, the repeated recovery of *P. aerogenes* supports its clinical relevance and makes a single accidental contamination less likely. However, because both organisms were concurrently recovered, the independent pathogenic contribution of *P. aerogenes* could not be definitively determined. In addition, the original culture plates were not retained for retrospective quantitative assessment; therefore, the relative abundance of *P. aerogenes* and *E. coli* in each culture could not be reliably compared. Accordingly, strain 21PA20240911 is interpreted as a repeatedly recovered, clinically significant isolate from a patient with symptomatic polymicrobial urinary tract infection, rather than a definitively established sole causative pathogen.

A total of 28 virulence-associated genes were identified in strain 21PA20240911, spanning multiple functional categories including adhesion, iron acquisition, LPS biosynthesis, and secretion systems. Notably, the presence of *tviB* and *wbpD* is particularly intriguing. The *tviB* gene, a component of the Vi capsular polysaccharide synthesis cluster in *Salmonella enterica serovar* Typhi, has rarely been reported in *Pasteurella* species (Wetter et al., 2012; Zhang et al., 2022). In other bacterial contexts, Vi capsular polysaccharide has been associated with protection against phagocytosis and complement-mediated killing. Therefore, the presence of a tviB-like gene in strain 21PA20240911 may indicate a potential virulence-associated feature; however, its function and contribution to immune evasion in this isolate remain to be experimentally determined. Similarly, *wbpD*, annotated as an O-antigen acetyltransferase, may be associated with lipopolysaccharide modification and serum resistance based on evidence from other bacterial species (Wenzel et al., 2005), but this role has not been verified in 21PA20240911. The restricted distribution of these two genes in our human clinical isolate and a duck-derived isolate from Sichuan suggests a distinct virulence-associated gene profile. Although free-range poultry farming is common in the patient’s rural residential environment in Hainan, additional genomic and epidemiological investigations are required to determine any potential epidemiological relationship. Subsequent functional studies are warranted to elucidate the role of these genes in the pathogenicity of *P. aerogenes*. Functional annotation further revealed 124 CAZymes, predominantly glycoside hydrolases and glycosyltransferases, indicating robust carbohydrate metabolism capacity (Drula et al., 2022), and 412 PHI-associated genes, supporting its genetic potential for host adaptation (Urban et al., 2025). These findings collectively suggest that strain 21PA20240911 possesses a comprehensive arsenal of virulence determinants and metabolic capabilities conducive to pathogenicity.

Strain 21PA20240911 was resistant to erythromycin among all tested agents. For several β-lactams (including penicillins), tetracyclines, and fluoroquinolones, MIC values exceeded the susceptible breakpoints defined for related *Pasteurellaceae* species; however, due to the absence of species-specific CLSI breakpoints for *P. aerogenes*, these results could only be described as “nonsusceptible” rather than “resistant”. For many other antibiotics, no CLSI breakpoints exist for any *Pasteurella* species, and only raw MIC values and the diameters of the inhibition zones could be presented.

Genomic analysis identified *tet (B)/tetR* and a PBP3/FtsI-like protein variant annotated by RGI/CARD against the *Haemophilus influenzae* PBP3 reference model, carrying D350N and S357N substitutions. These findings were consistent with the nonsusceptible phenotypes observed for tetracyclines and selected β-lactams, respectively (Bertram et al., 2022). Although beta-lactamase production was not detected by the nitrocefin-based test, PBP3/FtsI alterations may contribute to reduced β-lactam susceptibility by affecting penicillin-binding affinity (San Millan et al., 2011). This observation is consistent with reports that PBP3 alterations can be associated with reduced β-lactam susceptibility in *Pasteurellaceae* species.

Compared with swine-derived isolates from Thailand (Keeratikunakorn et al., 2024) and a poultry-derived isolate from Sichuan (Yuan et al., 2022), which exhibited broad resistance profiles, our clinical isolate also showed nonsusceptibility to multiple antimicrobial agents. This study suggests that it may be necessary for CLSI to consider establishing *Pasteurella*-specific breakpoints, as current criteria appear insufficient for capturing clinical resistance. Given the broad resistance observed across different sources, clinicians may benefit from relying on antimicrobial susceptibility testing rather than empirical therapy.

The separation of the *P. aerogenes*, *P. mairii*, and *A. rossii* genome groups observed in our ANI analysis was consistent with the taxonomic delineation of this complex reported by Christensen et al. (2005). Within this genomic framework, strain 21PA20240911 showed highest relatedness to *P. aerogenes* (Christensen et al., 2005).

The patient in this study resided in a rural area of Qionghai City, Hainan Province, where residents have the practice of raising free-range pigs and poultry around their dwellings. This living environment may have provided opportunities for exposure to *P. aerogenes*, as *P. aerogenes* is known to colonize the intestinal tract of swine and has been associated with reproductive disorders and abortions in animals, with previous reports of isolation from stillborn fetuses (Thorsen et al., 1994). Phylogenetic analysis placed strain 21PA20240911 in Clade II, together with multiple swine- and poultry-derived strains, and showed the closest genetic relatedness to isolates from Sichuan, China, which is consistent with, but does not prove, a potential zoonotic origin. Given that *P. aerogenes* is an opportunistic pathogen, healthcare workers should remain vigilant about this bacterium, particularly in susceptible populations including immunocompromised individuals, those with underlying diseases, and pregnant women. Enhanced awareness and surveillance are warranted to prevent and manage potential infections caused by this underrecognized zoonotic pathogen.

## Conclusion

In this study, we present the first complete genome sequence of a clinical *P. aerogenes* isolate from China. Strain 21PA20240911 showed a distinct virulence-associated gene profile, including the restricted occurrence of *tviB* and *wbpD*. Their functional roles in *P. aerogenes* warrant further investigation. Antimicrobial susceptibility testing showed that this isolate exhibited nonsusceptibility to multiple antimicrobial agents, and several resistance determinants were identified. These findings provide a genomic foundation for understanding the pathogenicity and antimicrobial resistance of this opportunistic pathogen. However, this study is based on a single isolate, and functional validation of the identified genes is needed. Future studies should focus on clinical recognition of this bacterium, rational antibiotic use guided by susceptibility testing, and health education for high-risk populations. From a One Health perspective, strengthening collaborative surveillance across humans, animals, and the environment is essential for preventing and controlling the spread of this pathogen. In conclusion, this study highlights the importance of continued surveillance of *P. aerogenes* in both clinical and veterinary settings.

## Data Availability

The datasets presented in this study can be found in online repositories. The names of the repository/repositories and accession number(s) can be found in the article/**Supplementary Material**.
