# Supplementary material for: Genomic and antimicrobial susceptibility characterization of a clinical Pasteurella aerogenes isolate from a human patient
Source: Front Cell Infect Microbiol. 2026 Sep 30;16:1900215. doi: 10.3389/fcimb.2026.1900215 (PMC13661337; doi:10.3389/fcimb.2026.1900215)
Supplement: Supplementary file 1 [file Table1.docx]

**Concise Methods for Comparative Genome Analysis**

# **Genome sequences and reference genome**

The complete chromosome of *Pasteurella aerogenes* strain 21PA20240911, a human clinical isolate, was assembled and annotated in-house (2,302,137 bp; GC content, 40.84%; 2,144 coding sequences [CDSs]). Species assignment was supported by 16S rRNA gene similarity (97.84% identity to *P. aerogenes* ATCC 27883; GenBank NR_118746.1). The complete genome of the type strain NCTC13378 (GCA_900637275.1/LR134362; 2,257,472 bp; GC content, 41.01%; 2,067 CDSs) was used as the comparative reference.

# **Functional annotation**

Protein-coding sequences from both genomes were annotated using eggNOG-mapper v2.1.2 against eggNOG v5.0.2, with DIAMOND v2.0.4 as the search engine. COG functional categories and KEGG pathway assignments were retrieved. COG annotations were obtained for 2,112 of 2,144 CDSs (98.5%) in 21PA20240911 and all 2,067 CDSs in NCTC13378. tRNA and rRNA genes were extracted from genome annotations; each genome contained 57 tRNA genes and 19 rRNA genes (six 16S, six 23S, and seven 5S).

# **Homologous regions and strain-specific genes**

Nucleotide homology was assessed by BLASTN (BLAST+ v2.15.0; E-value <= 1 x 10^-10) using 21PA20240911 as the query and NCTC13378 as the database. Of 377 detected segments, 296 were retained after removal of short, low-identity hits (<500 bp and <95% identity). Retained regions were assigned the COG category of overlapping CDSs; 230 COG-colored synteny ribbons were displayed.

Strain-specific genes were identified by reciprocal best-hit BLASTP analysis (E-value <= 1 x 10^-5). The highest-bitscore hit was retained for each query, and genes without a reciprocal best hit were defined as strain-specific. This analysis identified 241 genes unique to 21PA20240911 and 164 genes unique to NCTC13378.

# **Circos visualization**

A comparative circular genome map was generated with Circos v0.69-8 and exported as PNG and SVG. The chromosomes of 21PA20240911 (red) and NCTC13378 (blue) were displayed with tracks for GC content and GC skew (5-kb windows, 2-kb step), COG categories, KEGG pathway density (50-kb bins), tRNA and rRNA loci, defense-mechanism genes (COG V), strain-specific genes, the CRISPR array of 21PA20240911, and central BLASTN synteny ribbons. Ribbon color reflected the COG category of overlapping genes and transparency reflected sequence identity.

# **Data processing**

Data extraction and format conversion were performed using custom Python 3.8 scripts with Biopython v1.83. BLAST database construction and sequence searches were conducted with NCBI BLAST+ v2.15.0 (makeblastdb, blastn, and blastp).

# **Key software**

Circos v0.69-8; NCBI BLAST+ v2.15.0; eggNOG-mapper v2.1.2; eggNOG database v5.0.2; DIAMOND v2.0.4; Biopython v1.83; Python 3.8.

# **References**

Krzywinski M, Schein J, Birol I, et al. Circos: an information aesthetic for comparative genomics. Genome Research. 2009;19(9):1639-1645.

Camacho C, Coulouris G, Avagyan V, et al. BLAST+: architecture and applications. BMC Bioinformatics. 2009;10:421.

Cantalapiedra CP, Hernandez-Plaza A, Letunic I, et al. eggNOG-mapper v2: functional annotation, orthology assignments, and domain prediction at the metagenomic scale. Molecular Biology and Evolution. 2021;38(12):5825-5829.

Huerta-Cepas J, Szklarczyk D, Heller D, et al. eggNOG 5.0: a hierarchical orthology framework with improved functional annotations for eukaryotic, prokaryotic and viral sequences. Nucleic Acids Research. 2019;47(D1):D309-D314.

Buchfink B, Xie C, Huson DH. Fast and sensitive protein alignment using DIAMOND. Nature Methods. 2015;12(1):59-60.

Cock PJA, Antao T, Chang JT, et al. Biopython: freely available Python tools for computational molecular biology and bioinformatics. Bioinformatics. 2009;25(11):1422-1423.
